# Engineering processive DNA polymerases with maximum benefit at minimum cost

**DOI:** 10.3389/fmicb.2014.00380

**Published:** 2014-08-04

**Authors:** Linda J. Reha-Krantz, Sandra Woodgate, Myron F. Goodman

**Affiliations:** ^1^Department of Biological Sciences, University of AlbertaEdmonton, AB, Canada; ^2^Trevigen, Inc., Gaithersburg, MDUSA; ^3^University of Southern CaliforniaLos Angeles, CA, USA

**Keywords:** A+T- and G+C-rich DNA templates, DNA polymerase-DNA dynamics, DNA polymerase processivity, DNA polymerase translocation, DNA sequencing, motif A, pyrophosphorolysis, replication fidelity

## Abstract

DNA polymerases need to be engineered to achieve optimal performance for biotechnological applications, which often require high fidelity replication when using modified nucleotides and when replicating difficult DNA sequences. These tasks are achieved for the bacteriophage T4 DNA polymerase by replacing leucine with methionine in the highly conserved Motif A sequence (L412M). The costs are minimal. Although base substitution errors increase moderately, accuracy is maintained for templates with mono- and dinucleotide repeats while replication efficiency is enhanced. The L412M substitution increases intrinsic processivity and addition of phage T4 clamp and single-stranded DNA binding proteins further enhance the ability of the phage T4 L412M-DNA polymerase to replicate all types of difficult DNA sequences. Increased pyrophosphorolysis is a drawback of increased processivity, but pyrophosphorolysis is curbed by adding an inorganic pyrophosphatase or divalent metal cations, Mn^2+^ or Ca^2+^. In the absence of pyrophosphorolysis inhibitors, the T4 L412M-DNA polymerase catalyzed sequence-dependent pyrophosphorolysis under DNA sequencing conditions. The sequence specificity of the pyrophosphorolysis reaction provides insights into how the T4 DNA polymerase switches between nucleotide incorporation, pyrophosphorolysis and proofreading pathways. The L-to-M substitution was also tested in the yeast DNA polymerases delta and alpha. Because the mutant DNA polymerases displayed similar characteristics, we propose that amino acid substitutions in Motif A have the potential to increase processivity and to enhance performance in biotechnological applications. An underlying theme in this chapter is the use of genetic methods to identify mutant DNA polymerases with potential for use in current and future biotechnological applications.

## INTRODUCTION

The remarkable advances made in molecular biology in the last 40 years are dependent to a large extent on DNA polymerase-dependent methods. Researchers who pioneered the use of DNA polymerases for DNA sequencing, PCR and site-directed mutagenesis were awarded Nobel prizes for their ground-breaking achievements, but DNA polymerases play key roles in numerous additional indispensable methods including DNA labeling, cloning, whole genome amplification, and diagnostic techniques. Researchers, however, recognized from the earliest days that some DNA polymerase activities are counter-productive for *in vitro* applications. Nuclease activities, for example, can degrade primers and the newly synthesized DNA, yet some exonucleolytic proofreading activity is needed for high fidelity DNA replication. Other DNA polymerase activities need to be modified, for example the ability to utilize non-standard nucleotides and to replicate “difficult” DNA templates with simple repeats or sequences that are excessively rich in A+T or G+C base pairs. DNA polymerases from several organisms have been subject to extensive engineering in order to remove or curb unwanted activities and to modify or enhance others that are required for optimal performance *in vitro* (see several chapters in this volume and reviews by Hamilton et al., 2001; Reha-Krantz, 2008).

One of the most challenging tasks has been to engineer DNA polymerases to replicate difficult DNA sequences. DNA polymerases stall and often dissociate at difficult template sequences and these sequences are frequently sites for replication errors. For example (Streisinger et al., 1966; Streisinger and Owen, 1985) observed that simple repeat sequences in phage T4 genes are hotspots *in vivo* for frameshift mutations, which are typically insertions or deletions within mono- and di-nucleotide repeat sequences. The length of the repeat sequence affects the likelihood of frameshift mutations as hotspot mutation sites have longer repeat tracks than colder, less mutable sites. Streisinger et al. (1966) and Streisinger and Owen (1985) that frameshift mutations are created by transient separation of the primer and template strands followed by strand misalignment during reannealing to create an intermediate with an unpaired repeat in the template or primer strand, but with a correctly paired primer-template terminus that can be extended by a DNA polymerase. If the misaligned DNA strands are not corrected before the next round of chromosome replication, the repeat sequence will be expanded (if the unpaired repeat is in the primer strand) or contracted (if the unpaired repeat is in the template strand). As expected, these “slippery” DNA templates are also difficult to replicate *in vitro* as they slow or even prevent further replication and are sites for insertions and deletions (Rao, 1994; Fidalgo da Silva and Reha-Krantz, 2000; Clarke et al., 2001; Fazekas et al., 2010). Kunkel et al. (1994) demonstrated that +1 insertions in homopolymeric runs dramatically increase in reactions with the phage T7 DNA polymerase in the absence of thioredoxin, the T7 DNA polymerase processivity factor. Thus, decreased DNA polymerase processivity is correlated with increased strand misalignment mutagenesis, presumably by increasing DNA polymerase dissociation which provides an opportunity for the free primer-end to spontaneously denature and then to re-anneal in a misaligned configuration. This proposal is supported by other studies, for example, Fazekas et al. (2010).

We observed that the leucine to methionine (L412M) substitution in the conserved Motif A (**Table 1**) of the bacteriophage T4 DNA polymerase produced a mutant DNA polymerase that has improved ability to replicate difficult DNA sequences under typical DNA sequencing conditions. Furthermore, the L412M-DNA polymerase has increased ability to incorporate and extend modified nucleotides. Both activities are facilitated by increased intrinsic processivity (Reha-Krantz and Nonay, 1994), as explained below.

**Table 1:** Motif A in Phage T4 DNA polymerase and yeast DNA polymerases.

DNA pol	Motif A sequence	Phenotypes
[15pt] T4 wild type	408 D lt SLYPSII	Wild type
T4 L412M	M	Weak mutator^a^, strongly PAA-sensitive^a^
T4 I417V	V	Antimutator^a^, sensitive to reduced dGTP^a^
T4 L412M/I417V	M V	Antimutator^a^, PAA-sensitive
T4 L412I	I	Weak antimutator^a^, sensitive to reduced dGTP^a^
T4 S411T/L412M	TM	Weak antimutator^a^, sensitive to reduced dGTP^a^
		
Sc DNA pol δ	608 D fn SLYPSIM	Wildtype
Sc L612M δ	M	Mutator^b^, PAA-sensitive^b^
Sc L612M/V758Mδ^e,f^	M	Wildtype fidelity, PAA-resistant
		
Sc DNA pol α	864 D fn SLYPSII	Wildtype
Sc L868M-α	M	Mutator^c^, PAA-sensitive^b^
Sc L868F-α	F	Mutator^c^, PAA-sensitive^d^
Sc L868W-α	W	Weak mutator^c^, PAA-resistant^d^

*^a^ Data from **Table 2** and Reha-Krantz and Nonay (1994).*

*^b^ Data from Li et al. (2005); mutator phenotype observed in the absence of mismatch repair.*

*^c^ Data from Niimi et al. (2004).*

*^d^ Unpublished observations, LR-K.*

*^e^ V758M is near Motif C.*

*^f^Li (2004).*

The L412M substitution in the phage T4 DNA polymerase was identified by a genetic selection strategy as a second-site amino acid substitution that suppressed the excessive proofreading observed for several mutant DNA polymerases (Stocki et al., 1995; Li et al., 2010). As expected, the L412M substitution reduces proofreading activity, but base substitution mutations are increased moderately 10- to 40-fold, while frameshift mutations by only threefold at most (**Table 2**). Thus, high fidelity DNA replication observed for the wild type T4 DNA polymerase, about one error in 10^7^–10^8^ nucleotides incorporated (Kunkel et al., 1984), is essentially retained; hence, the T4 L412M-DNA polymerase is an excellent candidate polymerase for single-molecule sequencing and other applications where error-free replication is required. Note that amino acid substitutions in Motif A of DNA polymerases from several organisms have been observed to cause minor to major changes in replication fidelity, both increasing and decreasing accuracy (**Table 2**; Reha-Krantz and Nonay, 1994; Patel and Loeb, 2000; Patel et al., 2001; Fidalgo da Silva et al., 2002; Niimi et al., 2004; Li et al., 2005; Venkatesan et al., 2006, 2007; Pursell et al., 2007; Sakamoto et al., 2007; Nick McElhinny et al., 2008; Zhong et al., 2008).

**Table 2:** DNA replication fidelity by phage T4 wild type and mutant polymerases with amino acid substitutions in motif A.

DNA polymerases	Forward mutation frequency^a^	Types of ac mutations (percentage)	Base substitution reversion frequency^b^	Frameshift reversion frequency^c^
Wild type^d^	1/10^5^	GC→TA	(18%)	0.7/10^6^	1/10^6^
		AT→GC	(12%)		
		GC→TA		
		GC→CG	(3%)		
		AT→TA	(5%)		
		AT→CG	(1%)		
		Total base substitution	40%		
		Total frameshift	28%		
		Total other^e^	32%		

L412M^d^	35/10^5^	GC→AT	(37%)	9/10^6^	3/10^6^
		AT→GC	(2%)		
		GC→TA	(13%)		
		GC→CG	(2%)		
		AT→TA	(8%)		
		AT→CG	(9%)		
		Total base substitution	90%		
		Total frameshift	3%		
		Total other	7%		

I417V	nd^f^	nd		0.005/10^6,g^	nd
L412M/I417V	nd	nd		0.03/10^6,g^	nd
L412I	nd	nd		0.1/10^6,g^	nd
S411T/L412M	nd	nd		0.07/10^6,g^	nd

*^a^ Mutations that confer acriflavin resistance in the *ac* gene (Wang and Ripley, 1998).*

*^b^ Reversion of *rII*199*oc* to *rII*; mostly AT→GC mutations (Reha-Krantz, 1995).*

*^c^ Reversion of *rII*131 by +1 fs.*

*^d^ Data from several students, especially Alia Daoud and Fatima Kamal.*

*^e^ Total “other” are mostly deletions between direct repeats.*

*^f^ nd; not determined.*

*^g^ Data from Reha-Krantz and Nonay (1994).*

**FIGURE 1:**
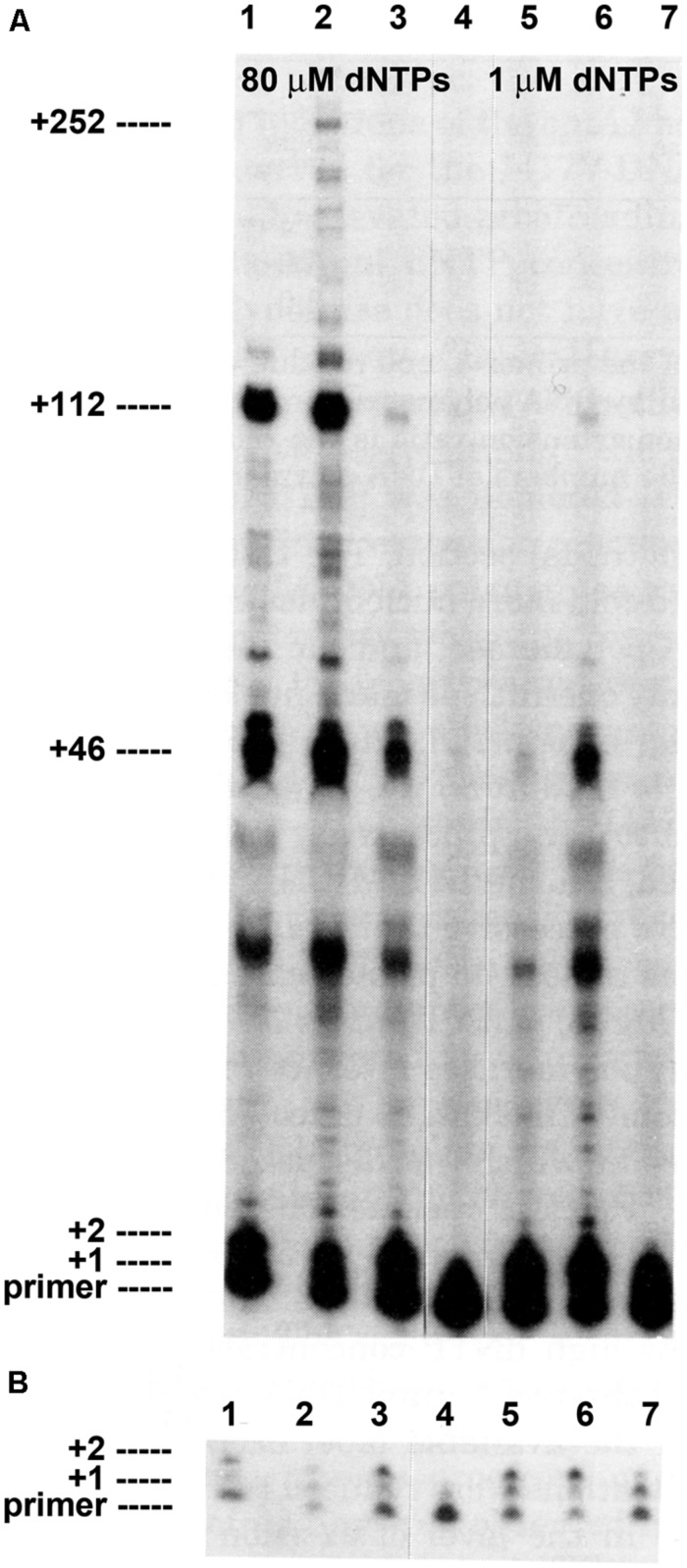
**Intrinsic processivity of bacteriophage T4 wild type and mutant DNA polymerases**. Primer elongation was determined in the presence of a heparin trap as described (see Materials and Methods). Reaction products were separated under denaturing gel electrophoresis conditions. **(A)** Almost no primer extension was detected if heparin was added before the DNA polymerase in the control reaction, lane 4. Significant primer extension was observed in reactions with 80 μM dNTPs: wild type T4 DNA polymerase, lane 1; L412M-DNA polymerase, lane 2; I417V-DNA polymerase, lane 3. Less primer extension was observed when dNTPs were reduced to 1 μM: wild type T4 DNA polymerase, lane 5; L412M-DNA polymerase, lane 6; I417V-DNA polymerase, lane 7. Prominent termination sites are indicated. **(B)** Short film exposure of the reactions in panel A. The figure is modified from Reha-Krantz and Nonay (1994) and is shown with permission from the ASBMB.

We review the development of the phage T4 L412M-DNA polymerase as an important DNA sequencing tool and its use in other applications. There are useful lessons to be learned because replication of difficult DNA templates remains salient with current DNA sequencing and amplification technologies. Because amino acid substitutions in Motif A of the bacteriophage T4 DNA polymerase have profound effects on intrinsic processivity (**Figure 1**), we propose that amino acid substitutions in Motif A of other DNA polymerases, especially the L-to-M substitution in family B DNA polymerases, have the potential to increase processivity and enhance DNA polymerase performance in biotechnological applications. A strategy to “evolve” mutant DNA polymerases with increased processivity and other desirable activities for *in vitro* applications (Bourn et al., 2011) is compared to our genetic evolution strategies. We also present new data about pyrophosphorolysis activity, a byproduct of increased intrinsic processivity. While the scope of this chapter is limited to methods used to enhance the intrinsic processivity of the phage T4 DNA polymerase, information about the naturally highly processive ϕ29 DNA polymerase is presented by Blanco et al. (1983) and Eid et al. (2009). The association of thioredoxin with the T7 DNA polymerase and the development of Sequenase^TM^ is presented by Zhu (2014). Increased processivity produced by fusing DNA-binding domains to DNA polymerases is discussed by Arezi et al. (2014) in this volume.

## MATERIALS AND METHODS

### DNA POLYMERASES AND DNA POLYMERASE ACCESSORY PROTEINS

Expression, purification, and characterization of wild type and mutant T4 DNA polymerases were described previously (Lin et al., 1987; Spicer et al., 1988; Reha-Krantz et al., 1993). The T4 gp45 clamp and clamp loading proteins (gp 44/gp62) and the T4 single-stranded DNA (ssDNA) binding protein (gp32) were expressed and purified as described (Shamoo et al., 1986; Rush et al., 1989), but with modifications for large scale production.

### DNA POLYMERASE INTRINSIC PROCESSIVITY

The intrinsic processivity of wild type and mutant phage T4 DNA polymerases was determined in the presence of a heparin trap as described by Reha-Krantz and Nonay (1994). Briefly, 20 μl reactions contained 25 mM HEPES, pH 7.5, 60 mM NaOAc, 1 mM dithiothreitol, 0.5 mM EDTA, 80 μM dNTPs, 0.2 mg/ml bovine serum albumin, 7.5 nM primed single-stranded circular M13 DNA (expressed as 3′-primer ends), and 150 nM DNA polymerase. Reactions were pre-incubated 5 min at 30°C and started by adding a solution of Mg^2+^ [Cf, 6 mM Mg(OAc)_2_] and heparin (Cf, 0.1 mg/ml). Reactions were incubated 15 s at 30°C and stopped by the addition of 2 μl 0.2 M EDTA. Reaction products were separated on DNA sequencing gels (7% acrylamide, 8 M urea) and the ^32^P-labeled products were visualized by exposure to Kodak X-Omat AR film.

### PYROPHOSPHOROLYSIS ASSAY CONDITIONS AND INHIBITION BY Mn^2+^ AND Ca^2+^ IONS

Pyrophosphorolysis activity was measured for the exonuclease-deficient D112A/E114A/L412M-DNA polymerase in 12 μl reactions containing partially digested duplex DNA that was 3′-labeled with [^32^P]dCMP (50 pmol of labeled 3′-ends/reaction), 10 nM DNA polymerase, 67 mM Tris-HCl (pH 7.5), 16.7 mM (NH_4_)_2_SO_4_, 0.5 mM DTT, 167 μg BSA/ml, 6.7 mM MgCl_2_, and 1 mM PPi. Reactions with Mn^2+^ or Ca^2+^ ions also contained 15 mM Na citrate. The PPi concentration varied from 0.5 to 6 mM and Mn^2+^ or Ca^2+^ ion concentrations varied from 0.25 to 10 mM. Reactions were stopped by the addition of 2 μl 0.2 M EDTA. The product of pyrophosphorolysis, [α-^32^P]dCTP, was separated by thin layer chromatography on polyethyleneimine impregnated cellulose (PEI) plates. Samples (2 μl) of the reactions were applied 1 cm from the bottom of the plate. The plates were developed in 50% ethanol up to 5 cm, dried and developed in 0.15 M KH_2_PO_4_ – 15% ethanol solvent until the solvent reached the top of the plate. Plates were dried and either the UV-absorbing spot was cut out and radioactivity was determined using a scintillation counter or the dried TLC plate was exposed to a PhosphorImager screen (Molecular Dynamics). These conditions were also studied under DNA sequencing conditions (see below).

Pyrophosphorolysis was also determined in assays using the fluorescence of the base analog, 2-aminopurine (2AP). The use of 2AP to study DNA polymerase function is described with detailed instruction by Reha-Krantz (2009). Briefly, 2AP fluorescence in DNA is quenched by base-stacking interactions, but the 2AP nucleotide is highly fluorescent. Thus, for primer-templates labeled at the 3′-end of a primer with 2AP, pyrophosphorolysis is detected as an increase in fluorescence intensity due to production of free 2AP deoxynucleoside triphosphate (d2APTP). Because 3′-exonuclease activity will also release the terminal 2AP nucleotide, but as the deoxynucleoside monophosphate (d2APMP), DNA polymerases were engineered to be exonuclease deficient with the D112A/E114A amino acid substitutions which prevent Mg^2+^ binding in the exonuclease active site (Reha-Krantz and Nonay, 1993; Elisseeva et al., 1999). Stopped-flow experiments were performed with the Applied Photophysics SX.18 MV spectrofluorometer. Samples were excited at 310 nm; a 335 nm cut-off filter was used. Temperature in the sample-handling unit was maintained at 20.0 ± 0.5°C. Reactions were initiated in the stopped flow by mixing equal volumes of a solution of 1400 nM exonuclease-deficient T4 DNA polymerase, 400 nM 2AP-DNA substrate, 5 mM PPi, 25 mM HEPES (pH 7.5), 2 mM DTT, 50 mM NaCl and 1 mM EDTA with a second solution of 16 mM MgCl_2_, 25 mM HEPES (pH 7.5), and 50 mM NaCl. After mixing, the final concentrations of reaction components were 700 nM exonuclease-deficient T4 DNA polymerase, 200 nM 2AP-DNA, 2.5 mM PPi, 8 mM MgCl_2_, 1 mM DTT, 25 mM HEPES (pH 7.5), 50 mM NaCl, and 0.5 mM EDTA. Between five and six determinations were performed for each reaction and mean values were calculated. The experimental traces were fit to either single or double exponential equations. The agreement of the curve fits was judged by analysis of the randomness of the distribution of residuals for curves generated by single or double exponential equations. Two DNA substrates were used that differed in the A+T- or G+C-richness in the primer-terminal region. A+T DNA: primer, 5′GCACGTCATCGGTAATP; template, 3′CGTGCAGTAGCCATTATGGATCGATGGTTT. G+C DNA: primer, 5′GCACGTCATTAACGGTP; template, 3′CGTGCAG TAATTGCCATGGATCGATGGTTT. The A+T and G+C primer terminal DNA sequences are indicated in bold font. P indicates 2AP.

### DNA SEQUENCING WITH THE T4 EXONUCLEASE-DEFICIENT, L412M-DNA POLYMERASE HOLOENZYME COMPLEX: FIDELITY^TM^

FIDELITY^TM^ (ONCOR, Gaithersburg, MD, USA) was a manual DNA sequencing kit that was marketed for general sequencing purposes but also for use to sequence difficult DNA templates. There are four steps. In the first step, primer (1 pmol), labeled at the 5′-end in reactions using T4 polynucleotide kinase and [γ^32^P]ATP, was annealed in a 10 μl reaction to 1 μg ssDNA M13 DNA in annealing buffer [25 mM Tris-HCl (pH 8.5), 20 mM MgCl_2_, and 50 mM NaCl]. Reactions were held at 65°C for 2 min and then cooled slowly over 30 min to 35°C. The reactions were then pulse-centrifuged and chilled briefly on ice. If the primer was not labeled, a second step was performed to label the primer DNA internally using [α^35^S] or [α^33^P]dATP at 1500 Ci/mmol, 10 mCi/ml. Internal labeling reactions contained the 10 μl annealed DNA produced in the first step; 3 μl T4 reaction buffer [0.4 M Tris-HCl (pH 8.5), 40 mM MgCl_2_, 40 mM DTT, 0.4 mg/ml acetylated BSA; 4 mM ATP; 1.4 μM each dTTP, dGTP, dCTP]; exonuclease-deficient L412M-DNA polymerase (10 nM); and water to produce a final total volume of 18 μl. The reaction was incubated at 40 to 42°C for 15 min and then placed on ice. Primer elongation with chain terminators was carried out in the third step. A mixture (6 μl) of T4 DNA polymerase accessory proteins (gp32, 2.7 mg/ml; gp44/62, 0.2 mg/ml; gp45, 0.7 mg/ml) in 20 mM Tris-HCl (pH 8.0), 100 mM NaCl, 1 mM EDTA, 1 mM DTT, and 62% glycerol was added to the 18 μl of labeled DNA and mixed. 5.5 μl of the elongation mix was added to 2 μl of the four separate termination reaction tubes. All termination mixes contained 300 μM dATP, dTTP, dGTP, and dCTP that were supplemented with 40 μM 3′NH_2_ddATP (A mix), or 80 μM 3′NH_2_ddTTP (T mix), or 40 μM 3′NH_2_ddGTP (G mix), or 80 μM 3′NH_2_ddCTP (C mix). The reactions were incubated at 40 to 42°C for 5 min. In the last (4th step), reactions were stopped and proteins degraded by addition of 5 μl of a solution containing 3 parts Stop Solution (95% formamide, 20 mM EDTA, 0.05% bromophenol blue, 0.05% xylene cyanol blue) and 1 part Proteinase K Solution (100 μg/ml proteinase K in 50 mM Tris-HCl, pH 8.0). The reactions were mixed and incubated at 40°C for 15 min. Samples were heated at 80°C immediately prior to loading samples for gel electrophoresis.

Note, for reactions with inorganic pyrophosphatase (iPPase), the T4 DNA polymerase solution also contained 8 U/ml yeast iPPase. Alternatively, pyrophosphorolysis was inhibited in reactions with 16.7 mM Mg^2+^, 15 mM Na citrate and either 2.5 mM Mn^2+^ or 10 mM Ca^2+^.

## RESULTS

### BIOCHEMICAL CHARACTERIZATION OF THE BACTERIOPHAGE T4 L412M-DNA POLYMERASE

#### Increased intrinsic processivity

DNA polymerase intrinsic processivity refers to the number of nucleotides incorporated during one enzyme-DNA association replication cycle, but intrinsic processivity is partially masked *in vivo* by the association of processivity factors. For many DNA polymerases that function at replication forks, a clamp protein (gp45 in phage T4, PCNA in eukaryotes and the beta sliding clamp in *Escherichia coli*) tethers the DNA polymerase to the DNA template. The gp45 and PCNA clamps are composed of three identical subunits that form a doughnut structure around duplex DNA. The clamp is proposed to function as a tool belt to tether one or more DNA polymerases or other proteins found at replication forks (Pagès and Fuchs, 2002; Indiani et al., 2005; Moldovan et al., 2007). Although researchers have known for years that tethering of the T4 DNA polymerase in holoenzyme complexes increases both polymerase and exonuclease activities, Yang et al. (2004) observed that the primer-template appears to transfer between two tethered polymerases during proofreading. Thus, apparent continuous replication by the tethered T4 DNA polymerase may involve coordinated replication by two DNA polymerases tethered to the same clamp (Joyce, 2004). Tethering, however, does not fully compensate for differences in intrinsic processivity as explained below.

Without tethering, the wild type T4 DNA polymerase has little ability to initiate or extend a primer (**Figure 1**; Reha-Krantz and Nonay, 1993, 1994; Spacciapoli and Nossal, 1994). In reactions with ^32^P-labeled primer, single-stranded circular DNA and heparin to trap dissociated DNA polymerase, the vast majority of primers are not extended by the wild type T4 DNA polymerase and the few extensions detected are limited to incorporation of one or two nucleotides (**Figure 1B**, lane 1). Longer extension products are visible with prolonged film exposure (**Figure 1A**, lane 1), which means that most non-tethered T4 DNA polymerase molecules cannot extend the primer, the few complexes that are competent for extension incorporate just one or two nucleotides before dissociation, and only a few of these complexes escape the initiation phase and commence primer elongation. However, for primer extension complexes that escape early dissociation, there are preferential termination sites as indicated by discrete bands after incorporation of 46, 112, and 252 nucleotides (**Figure 1A**). Thus, the intrinsic processivity of the wild type T4 DNA changes from an initial very low or essentially non-processive state to a state with higher processivity during the elongation phase, but the elongating DNA polymerase remains sensitive to difficult DNA sequences as demonstrated by preferential termination sites. The role of the clamp then is in initiation and in assisting replication through difficult DNA sequences.

Amino acid substitutions in Motif A of the T4 DNA polymerase (**Table 1**) dramatically alter intrinsic processivity. Longer extension products are visible for the L412M-DNA polymerase (**Figure 1A**, lane 2), especially when concentrations of deoxyribonucleoside triphosphates (dNTPs) are reduced to 1 μM (**Figure 1**, lane 6); thus, more L412M-DNA polymerase complexes are able to escape the initiation phase and to replicate past difficult sequences. Kinetic studies are consistent with these observations. In standing start primer extension assays, the *K*_d_ for dAMP incorporation is lower for the L412M-DNA polymerase compared to wild type, 11 versus 16 μM (Hariharan et al., 2006), and the *k*_off_ rate is about twofold slower (Fidalgo da Silva et al., 2002). While the L412M substitution is the most conservative substitution, dramatic changes in the opposite direction are observed for the slightly less conservative L412I substitution. The L412I-DNA polymerase cannot replicate DNA when the dGTP pool is reduced and an antimutator phenotype is observed instead of the mutator phenotype observed for the L412M-DNA polymerase (**Table 1**). These features resemble the consequences of another conservative substitution in Motif A, I417V. The I417V-DNA polymerase is much less processive than the wild type T4 DNA polymerase (**Figure 1**, lane 3), especially when dNTP pools are reduced (**Figure 1**, lane 7). Like the L412I-DNA polymerase, the I417V-DNA polymerase cannot replicate DNA when the dGTP pool is reduced (Reha-Krantz and Nonay, 1994; Stocki et al., 1995) and proofreading is increased as indicated by the antimutator phenotype (**Table 2**). The low processivity of the I417V-DNA polymerase means that replication is inhibited by subtle DNA damage, for example a phosphotriester, which does not impede replication by other DNA polymerases (Tsujikawa et al., 2003). Because the differences in replication fidelity and sensitivity to dNTP pools are observed *in vivo* for the mutant DNA polymerases in the presence of the gp45 clamp, intrinsic processivity prevails in the presence of tethering and determines the stability of DNA polymerase complexes when dNTP pools are low, when DNA templates are damaged or difficult sequences are present, and if proofreading will be initiated.

Sequences at preferential termination sites are informative because they identify DNA sequences that are difficult for the T4 DNA polymerase to replicate. Many termination sites occur at simple repeats: the +46 product terminates within the template ATAT sequence and the +112 product terminates at the beginning of the template GCGC sequence (**Figure 1**; Reha-Krantz and Nonay, 1993). Termination with another DNA template was observed at the beginning of a template CACACA sequence (Spacciapoli and Nossal, 1994). These dinucleotide repeat termination sites are distinct from another type of difficult sequence, namely hairpin DNA structures that form between inverted repeats in ssDNA ahead of an advancing DNA polymerase. The T4 DNA polymerase has little ability to bypass hairpin structures in the absence of T4 ssDNA binding protein (gp32) and the clamp (gp45) and clamp loading proteins (Huang et al., 1981; Roth et al., 1982; Bedinger et al., 1989; Jarvis et al., 1991; Hacker and Alberts, 1994).

The simple repeat sequences at the termination sites in **Figure 1** likely cause pausing, which causes enzyme dissociation that is associated with initiation of the proofreading pathway (see review by Reha-Krantz, 2010). While mismatches at the primer-end trigger proofreading as an error avoidance mechanism (Brutlag and Kornberg, 1972; Muzyczka et al., 1972), gratuitous proofreading of correctly paired primer ends can be caused by anything that hinders continued primer elongation. In contrast to dissociation of the T4 DNA polymerase when proofreading is initiated, return of the trimmed primer-end to the polymerase active site is rapid and processive, even in the absence of tethering (Reddy et al., 1992; Fidalgo da Silva and Reha-Krantz, 2007). With respect to *in vitro* applications, less gratuitous proofreading and subsequent dissociation is observed for the T4 L412M-DNA polymerase than for other DNA polymerases; hence, difficult template sequences, low dNTP concentrations, and other factors that impede nucleotide incorporation will be less problematic for the more processive DNA polymerase.

#### Biochemical mechanism for increased intrinsic processivity by the T4 L412M-DNA polymerase; insights provided by heightened sensitivity to PAA

**FIGURE 2:**
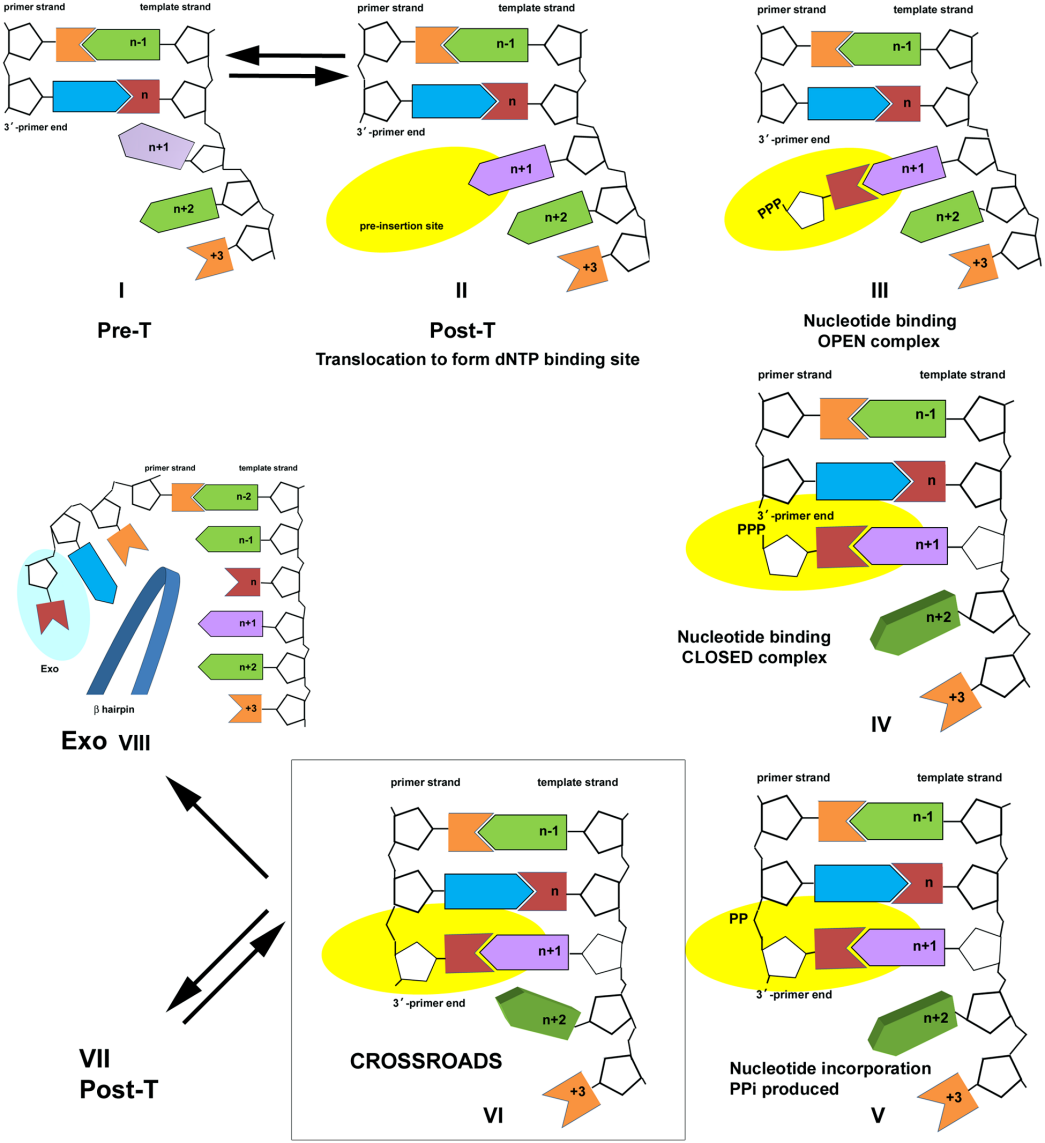
**Nucleotide incorporation scheme for the bacteriophage T4 DNA polymerase**. Pre- and Post-T complexes are in equilibrium (Complexes I and II). dNTP binding results in formation of an open ternary complex (Complex III); a conformation change to evaluate the accuracy of the incoming nucleotide results in formation of the closed ternary complex (Complex IV). Chemistry takes place to join the incoming nucleotide to the primer end and the PPi byproduct is formed (Complex V). PPi dissociates to form Complex VI, a new Pre-T, which is at the crossroads of three pathways. Complex VI is in equilibrium with the Post-T complex (Complex VII); binding the correct nucleotide starts another cycle of nucleotide incorporation. Alternatively, PPi may bind to Complex VI to form Complex V, which leads to the reverse reaction (pyrophosphorolysis). PAA, as a PPi mimic, also binds to Complex VI. A third alternative is proofreading (Complex VIII). The figure is modified from Reha-Krantz et al. (2011) and is shown with permission from the ACS.

The L412M substitution converts the T4 DNA polymerase from resistance to sensitivity to the herpes virus antiviral drug, phosphonoacetic acid (PAA). PAA-sensitivity is observed for the T4 L412M-DNA polymerase *in vivo* (**Table 1**; Reha-Krantz et al., 1993; Li et al., 2010) and *in vitro* (Reha-Krantz and Nonay, 1994). PAA resembles pyrophosphate (PPi), a byproduct of nucleotide incorporation; hence, PAA-sensitivity indicates increased PPi binding. PAA-sensitivity can be understood in terms of the nucleotide incorporation pathway described in **Figure 2** (Reha-Krantz et al., 2011). Complex I is a pre-translocation (Pre-T) complex. Complex II is formed by translocation forward by one template position, which removes the PPi binding site present in Complex I and creates a new nucleotide binding pocket (pre-insertion site). Complexes I and II are in rapid equilibrium (Fidalgo da Silva et al., 2002; Hariharan et al., 2006). Nucleotide binding produces the open ternary complex, Complex III, and a conformational change produces the closed ternary complex, Complex IV. Nucleotide incorporation takes place in Complex V; then PPi is released to form Complex VI, a Pre-T complex like Complex I except that the primer-end has been extended by one nucleotide.

Complex VI is at the crossroads of three competing pathways. The DNA polymerase may continue nucleotide incorporation by translocating to form Complex VII, a new post-T complex, or the reverse reaction can be initiated by re-binding PPi to form Complex V and then catalyzing pyrophosphorolysis, which produces dNTP and shortens the primer strand by one nucleotide (Complex IV). This is the pathway that is sensitive to PPi-like antiviral drugs. A third possibility is proofreading, which is initiated if the primer-end is mismatched, but gratuitous proofreading is also possible if polymerization conditions are not optimal, for example low dNTP pools. For proofreading, the end of the primer strand is separated from the template and transferred to the exonuclease site. A beta hairpin structure located in the exonuclease domain of the T4 DNA polymerase acts as a wedge to stabilize the exonuclease complex, Complex VIII (Stocki et al., 1995; Marquez and Reha-Krantz, 1996; Reha-Krantz, 1998, 2010; Reha-Krantz et al., 1998; Hogg et al., 2004, 2007; Subuddhi et al., 2008). All three pathways are in competition, but differences in DNA polymerase interactions with the primer-template affect which pathway is chosen. PAA-sensitivity indicates that the T4 L412M-DNA polymerase favors forming the Pre-T complex, which has a PPi-binding site, over the Post-T complex, which has a dNTP binding site. Although PPi-like antiviral drugs could conceivably inhibit replication by interfering with dNTP binding in Post-T complexes, structural studies show that PPi-like phosphonoformic acid (PFA) traps a mutant RB69 DNA polymerase in a complex that resembles Complex V (Zahn et al., 2011) as expected if PFA binds to the Pre-T complex.

Formation of Pre- and Post-T complexes can also be observed in assays that use the fluorescence of the base analog 2AP. Pre-T complexes have higher fluorescence intensity than Post-T complexes for DNA substrates with 2AP positioned in the +1 position in the template strand, adjacent to the terminal base pair (Mandal et al., 2002; Hariharan and Reha-Krantz, 2005; Reha-Krantz et al., 2011). The higher fluorescence intensity of polymerase complexes formed with the L412M-DNA polymerase indicates that the L412M-DNA polymerase favors formation of the highly fluorescent Pre-T complexes over Post-T complexes in the absence of PPi and dNTPs (Hariharan and Reha-Krantz, 2005). The role of the Pre-T complex in drug sensitivity is further substantiated by studies that show that PFA inhibits the HIV-1 reverse transcriptase by trapping a Pre-T complex (Marchand et al., 2007). We extend these findings by linking PAA-sensitivity of the L412M-DNA polymerase to increased intrinsic processivity, which suggests that Pre-T complexes are more stable, and less subject to enzyme dissociation than Post-T complexes (see further discussion by Li et al., 2010).

#### Increased pyrophosphorolysis is observed for the T4 L412M-DNA polymerase

Increased pyrophosphorolysis is expected for PAA-sensitive DNA polymerases because the equilibrium between Pre- and Post-T complexes favors Pre-T complexes, which can bind PPi (**Figure 2**). The PAA-sensitive T4 L412M-DNA polymerase catalyzes a robust pyrophosphorolysis reaction. Section “Materials and Methods” for assay conditions. The pyrophosophorolysis rate in the presence of 2.5 mM PPi for the exonuclease-deficient (D112A/E114A) L412M-DNA polymerase is fivefold higher than the rate detected for the exonuclease-deficient T4 DNA polymerase with the wild type Motif A sequence and more than 80-fold higher than the less processive, exonuclease-deficient I417V-DNA polymerase. The *K_m_* for PPi for the exonuclease-deficient L412M-DNA polymerase was ∼0.5 mM compared to 2 mM for the exonuclease-deficient DNA polymerase with the wild type Motif A and was too high to be measured for the exonuclease-deficient I417V-DNA polymerase (Damaraju and Reha-Krantz, unpublished observations).

#### Mechanisms to curb pyrophosphorolysis catalyzed by the L412M-DNA polymerase; formation of divalent cation-PPi complexes

**FIGURE 3:**
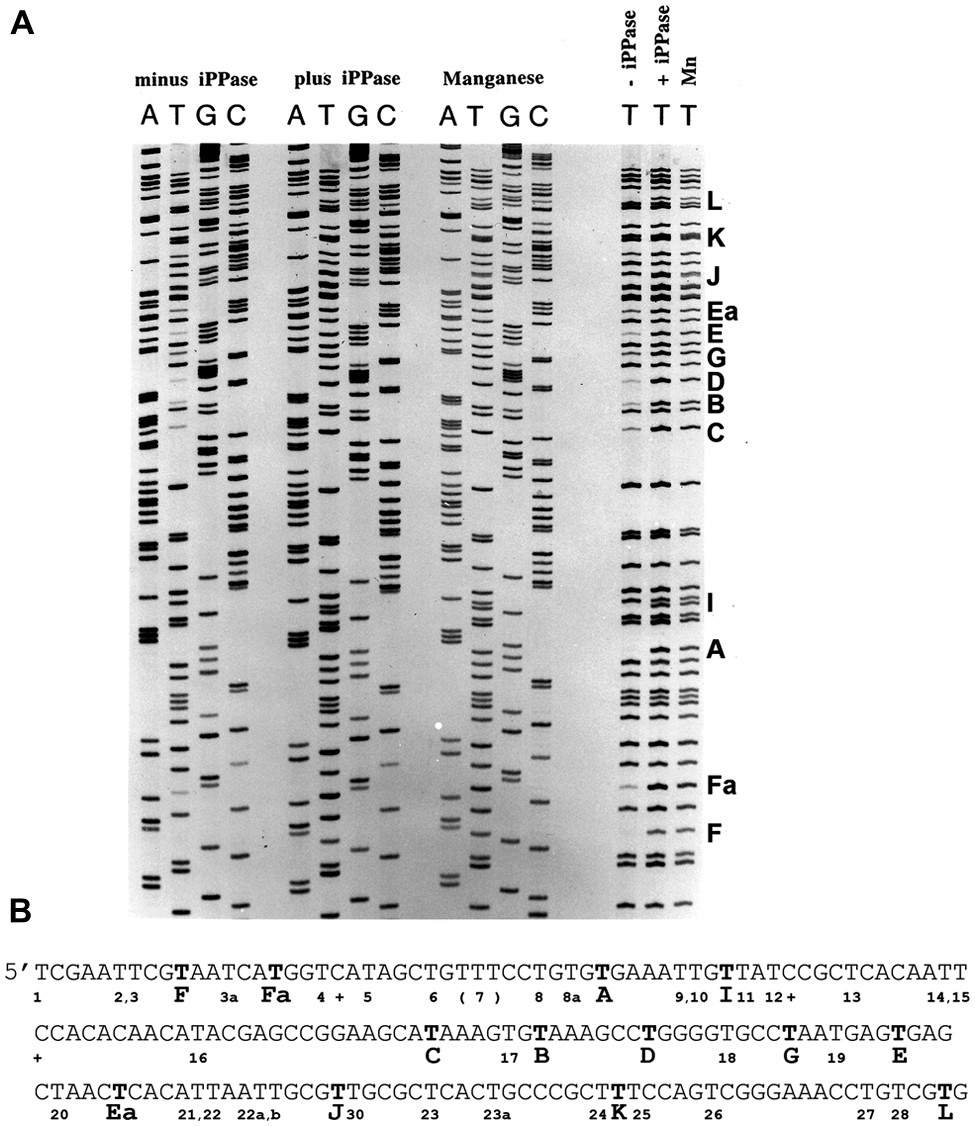
**Pyrophosphorolysis detected in DNA sequencing reactions with FIDELITY^TM^**. **(A)** Three sets of DNA sequencing reactions were performed (see Materials and Methods): without a pyrophosphorolysis inhibitor (minus iPPase), with added yeast inorganic pyrophosphatase (plus iPPase), and with manganese. Results similar to the manganese supplemented reactions were detected with calcium. No or faint bands were detected for several products terminating in T in the minus iPPase reactions. Reactions terminating in T for all sets of reactions are shown to the right; missing or faint reaction products terminating in T are indicated by a capital letter to the right of the lanes. **(B)** The DNA sequence for the reactions shown in panel A. Bold Ts indicate missing or faint terminating Ts; the letter code identifies pyrophosphorolysis sensitive sites. The sequences are analyzed in **Table 3**. Pyrophosphorolysis-resistant Ts are indicated by a number below the sequence.

Pyrophosphorolysis is the reversal of the polymerization reaction, but pyrophosphorolysis is usually not a problem during DNA replication *in vivo* because iPPase degrade PPi to prevent build-up of the high concentrations of PPi needed to drive pyrophosphorolysis. But high concentrations of PPi formed during DNA sequencing reactions support pyrophosphorolysis, which results in the degradation of chain-terminated sequencing products and ambiguous sequencing data (**Figure 3**). The addition of iPPase to sequencing reactions is often used to prevent pyrophosphorolysis (Tabor and Richardson, 1990), and this is successful in sequencing reactions catalyzed by the L412M-DNA polymerase, but divalent metal ions – Mn^2+^ and Ca^2+^, are equally effective when combined with Mg^2+^ in the presence of 15 mM Na citrate (**Figure 3**; Damaraju and Reha-Krantz, unpublished observations).

The mechanism of inhibition of pyrophosphorolysis by divalent metal cations is complex because, as demonstrated for yeast iPPase (Ridlington and Butler, 1972), while some divalent metal ions such as Mg^2+^ are needed for catalysis (activators), other metal ions such as Mn^2+^ or Ca^2+^ may inhibit activity by binding to the enzyme or DNA or by forming complexes with PPi. We observed this complexity for the nucleotide incorporation and pyrophosphorolysis reactions catalyzed by the T4 exonuclease-deficient L412M-DNA polymerase (Damaraju and Reha-Krantz, unpublished data). Either Mg^2+^ or Mn^2+^ can support nucleotide incorporation and pyrophosphorolysis reactions, but Mg^2+^ supports both reactions optimally at concentrations from 1 to >5 mM, while Mn^2+^ is only half as effective as Mg^2+^ and over a narrow concentration range that peaks at about 0.5 mM. In the presence of 15 mM Na citrate, however, Mn^2+^ supports nucleotide incorporation over a broad concentration range up to 5 mM, but the pyrophosphorolysis reaction is supported only over the narrow range from 0.5 to 1 mM. Thus, Na citrate protects the nucleotide incorporation reaction at high concentrations of Mn^2+^, but not the pyrophosphorolysis reaction. This means that pyrophosphorolysis catalyzed DNA degradation can be avoided in DNA sequencing reactions by the addition of 15 mM Na citrate and 3 to 5 mM Mn^2+^, but note further improvements discussed below.

**FIGURE 4:**
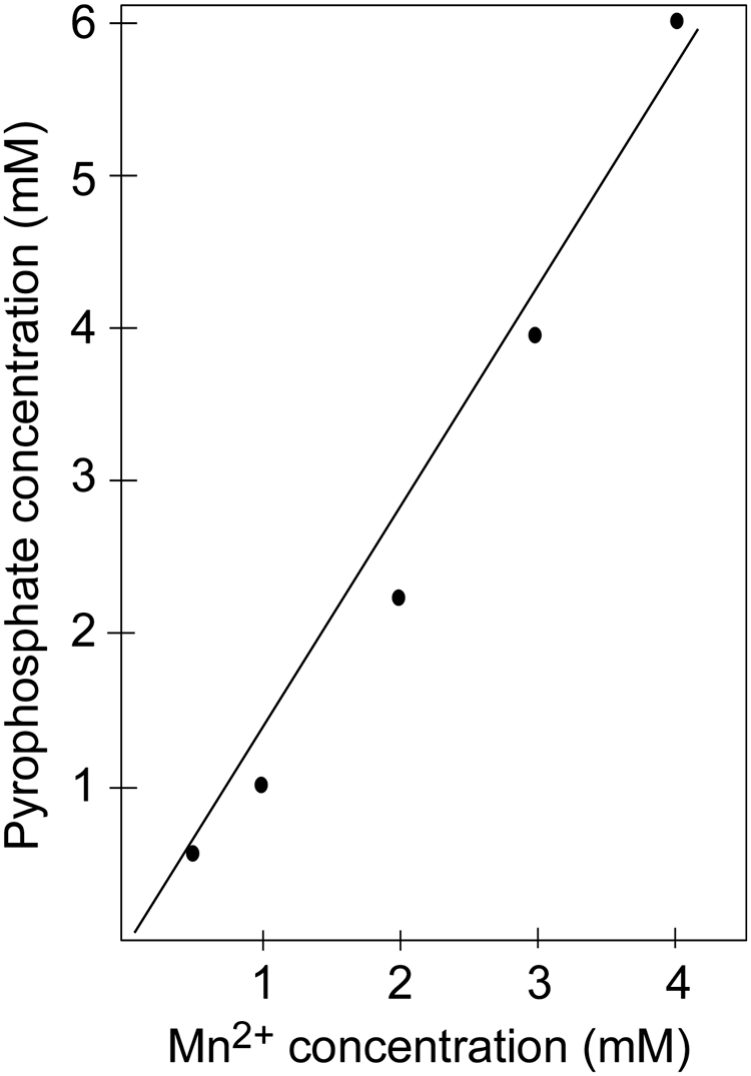
**Inhibiting pyrophosphorolysis with manganese**. Increased manganese is needed to inhibit the pyrophosphorolysis reaction catalyzed by the exonuclease-deficient L412M-DNA polymerase when the concentration of PPi is increased.

Na citrate protects the nucleotide incorporation reaction by functioning as a weak chelator, which reversibly binds divalent metal cations; however, PPi also forms complexes with metal cations. This point is demonstrated by the observation that higher amounts of Mn^2+^ ions are needed to inhibit pyrophosphorolysis by the T4 L412M-DNA polymerase as the concentration of PPi is increased (Damaraju and Reha-Krantz). For example, in the presence of 1 mM PPi, ∼ 1 mM Mn^2+^ produces 50% inhibition of pyrophosphorolysis; for 2 mM PPi, ∼2 mM Mn^2+^ is needed for 50% inhibition; and for 4 mM PPi, >3 mM Mn^2+^ is required to achieve the same level of inhibition (**Figure 4**). The stoichiometry of the reaction is consistent with formation of Mn-PPi chelate complexes or in the case of Ca^2+^, Ca-PPi chelate complexes. Thus, there is a complex equilibria involving one or more metal ions (Me): DNA pol-Me, citrate-Me, PPi- Me, free Me^2+^, free DNA pol, free PPi, free Na citrate, and active and inactive DNA pol- Me-PPi complexes. While Mn-PPi binding may inactivate pyrophosphorolysis directly, this is not the most likely explanation for inhibition in reactions with Na citrate and Mn^2+^ above 3 mM because nucleotide incorporation is not inhibited; Mn-PPi binding would be expected to inhibit both reactions. Another explanation is that Mn^2+^ sequesters PPi and reduces the high concentration of PPi needed for the pyrophosphorolysis reaction, but there is sufficient free Mn^2+^ (∼0.5 mM) to support, but not to inhibit, the nucleotide incorporation reaction. The ability of Na citrate to maintain low concentrations of Mn^2+^ has been proposed by others (Beckman et al., 1985; Tabor and Richardson, 1989, 1990), but in this case Mn^2+^ was thought to improve the evenness of band intensities (DNA sequencing products) by reducing discrimination against chain-terminating nucleotides. Ca^2+^ can also chelate PPi and effectively prevent pyrophosphorolysis (results not shown).

Although conditions were optimized for Mn^2+^-dependent nucleotide incorporation and minimal pyrophosphorolysis, nucleotide incorporation was only 60% of what is observed in Mg^2+^-dependent reactions and Ca^2+^ does not support the nucleotide incorporation reaction catalyzed by T4 DNA polymerase. Optimal nucleotide incorporation and minimal pyrophosphorolysis activity were obtained in mixed, divalent metal reactions with Mg^2+^ and either Mn^2+^ or Ca^2+^ in the presence of Na citrate. For DNA sequencing using FIDELITY^TM^ (see Materials and Methods), pyrophosphorolysis was essentially eliminated in reactions with 16.7 mM Mg^2+^, 15 mM Na citrate and either 2.5 mM Mn^2+^, or 10 mM Ca^2+^ without adverse effects on DNA sequencing efficiency (**Figure 3**).

#### DNA sequence context effects on the pyrophosphorolysis reaction catalyzed by the T4 L412M-DNA polymerase

DNA sequence effects have been reported for the pyrophosphorolysis reactions catalyzed by the Klenow fragment of *E. coli* DNA polymerase I (Mizrahi et al., 1986) and the phage T7 DNA polymerase (Tabor and Richardson, 1990), but systematic studies were not carried out to determine if specific DNA sequences are required for pyrophosphorolysis. We observed both general and specific DNA sequence contexts for the pyrophosphorolysis reactions catalyzed by the exonuclease-deficient T4 wild type and L412M-DNA polymerases.

Conditions that favor formation of Pre-T complexes (Complex VI, **Figure 2**) are predicted to increase pyrophosphorolysis because PPi can bind to this complex. In previous studies using the fluorescence of the base analog, 2AP, more Pre-T than Post-T complexes are formed with A+T-rich than G+C-rich DNA substrates (Hariharan and Reha-Krantz, 2005; Hariharan et al., 2006). Thus, pyrophosphorolysis is predicted to favor DNA substrates with A+T-rich primer-terminal regions. This was observed in reactions with A+T- and G+C-rich DNAs labeled with 2AP at the 3′-primer end (see Materials and Methods). The rate for release of the terminal d2APTP by the exonuclease-deficient L412M-DNA polymerase in the presence of 2.5 mM PPi was sixfold faster for the A+T-rich DNA compared to the G+C-rich DNA, 14 s^-1^ compared to 2.4 s^-1^ (Damaraju and Reha-Krantz). The exonuclease-deficient T4 DNA polymerase with the wild type Motif A sequence also favored A+T-rich DNA, but with slower rates, 3 and 0.5 s^-1^ for A+T- and G+C-rich DNAs, respectively. However, while pyrophosphorolysis was observed for DNA substrates with a terminal 2AP opposite template T, pyrophosphorolysis was not detected for 2AP opposite template C as expected if pyrophosphorolysis requires a matched primer-end. DNA substrates with A+T-rich primer-terminal regions also favor the exonucleolytic proofreading pathway (Bessman and Reha-Krantz, 1977; Bloom et al., 1994; Reha-Krantz, 2010). Thus, increased formation of Pre-T complexes, which likely reflects decreased ability to form Post-T complexes, increases the likelihood of proofreading or pyrophosphorolysis. Proofreading is favored if the primer-end is mismatched and pyrophosphorolysis is observed if sufficient PPi is present.

Specific DNA sequences were observed to promote pyrophosphorolysis under DNA sequencing conditions using the FIDELITY^TM^ manual DNA sequencing kit developed by ONCOR, Gaithersburg, MD, USA. Reactions contained the exonuclease deficient L412M-DNA polymerase, the gp45 processivity factor, the gp45 loading proteins as well as the T4 ssDNA binding protein, gp32, to prevent formation of DNA hairpin structures in ssDNA. Reaction products were internally labeled with α^33^P-dATP; the template was single-stranded circular DNA. Instead of 2′,3′-dideoxy nucleoside triphosphate terminators (ddNTPs), 3′-amino-2′,3′-dideoxy nucleoside triphosphates (3′NH_2_ddNTPs) were used because the T4 DNA polymerase showed less discrimination. 3′NH_2_ddNTPs can be purchased from TriLink BioTechnologies, San Diego, CA, USA. All reaction components and procedures are described in Section “Materials and Methods.” Four separate reactions were run, each with a different chain-terminator. The reaction products were separated by polyacrylamide gel electrophoresis under denaturing conditions (**Figure 3A**). In the absence of agents that reduce pyrophosphorolysis – iPPase or manganese (Mn^2+^), there are missing bands due to pyrophosphorolysis as well as many faint bands (**Figures 3A,B**).

It is readily apparent that not all chain-terminated primer termini are equally sensitive to pyrophosphorolysis; only primer ends ending in “T” were subject to pyrophosphorolysis, but not all terminal Ts (see right side of **Figure 3A**). Examination of 200 nucleotides of DNA sequence (**Figure 3B**) with 54 sequencing products that terminated in T revealed that only 12 of the terminal Ts were subject to pyrophosphorolysis. What is different about the DNA sequences for pyrophosphorolysis-sensitive and insensitive sites? There are three related sequences in the primer-template region that promote pyrophosphorolysis (**Table 3**); the sequences differ only in the base pairs at the -1 and -2 positions. Sequence 1 has a G_template_C_primer_ base pair in the -2 position; sequence 2 has a C_template_G_primer_ base pair in the -1 position, but not in the -2 position; and sequence 1/2 has a G_template_C_primer_ base pair in the -2 and a C_template_G_primer_ base pair in the -1 position. A template “G” was never observed in the +1 position in the template strand, even though statistically 2 or 3 were expected in the 11 sequences examined. Note that a “G” in the +1 position for a terminal T at position 26 in the DNA sequence (**Figure 3B**) stabilized the T to pyophosphorlysis even though the remainder of the consensus sequence is present. Similarly, a template “A” was not observed in the +4 position, but an A stabilized the Ts at DNA sequence positions 5 and 8. The consensus sequences 1, 2 and 1/2 (**Table 3**) are required to sensitize terminal Ts to pyrophosphorolysis, but one exception was observed, see Ea in **Figure 3**. Although the consensus sequence is not present, the terminal T is just before a dinucleotide GT repeat, which may stall formation of Post T complexes and, thus, promote pyrophosphorolysis. Thus, the pyrophosphorolysis sensitive sites observed in **Figure 3** may be an underestimation of the number of sensitive sites. Nevertheless, the strong bias for pyrophosphorolysis for only primers ending in “T” and only for a subset of terminal “Ts” indicates sequence specificity for the pyrophosphorolysis reaction catalyzed by the T4 L412M-DNA polymerase.

**Table 3:** DNA sequences that promote pyrophosphorolysis by the phage T4 L412M-DNA polymerase.

Sequence		Primer-template									
		–4	–3	–2	–2	–1	n^a^	+1	+2	+3	+4
1	**Template**	**X^b^**	**A/C**	**G**	**X**	**A**	**Not**	**G**	**X**	**X**	**Not**	**A**
	**Primer**	**X**	**T/G**	**C**	**X**	**T^c^**				
	**Fa^d^**	T	**A**	**G**	T	**A**	C	C	A	G
		A	**T**	**C**	A	**T**				
	**C**	T	**C**	**G**	T	**A**	T	T	T	C
	**D**	A	**C**	**G**	G	**A**	C	C	C	C
		A	**G**	**C**	A	**T**				
	**G**	A	**C**	**G**	G	**A**	T	T	A	C
		T	**G**	**C**	C	**T**				
	**K**	G	**C**	**G**	A	**A**	A	G	G	T
		C	**G**	**C**	T	**T**				
2	**Template**	**X**	**A/C**	**A/T**	**C**	**A**	**Not**	**G**	**X**	**X**	**Not**	**A**
	**Primer**	**X**	**T/G**	**T/A**	**G**	**T**				
	**A**	A	**C**	**A**	**C**	**A**	C	T	T	T
		T	**G**	**T**	**G**	**T**				
	**I**	T	**A**	**A**	**C**	**A**	A	T	A	G
		A	**T**	**T**	**G**	**T**				
	**B**	T	**C**	**A**	**C**	**A**	T	T	T	C
		A	G	T	G	T				
	**E**	A	**C**	**T**	**C**	**A**	C	T	C	G
		T	**G**	**A**	**G**	**T**				
1/2	**Template**	**X**	**A/C**	**G**	**C**	**A**	**Not**	**G**	**X**	**X**	**Not**	**A**
	**Primer**	**X**	**T/G**	**C**	**G**	**T**				
	**F**	A	**A**	**G**	**C**	**A**	T	T	A	G
		T	**T**	**C**	**G**	**T**				
	**J**	A	**C**	**G**	**C**	**A**	C	G	C	G
		T	**G**	**C**	**G**	**T**				
	**L**	C	**A**	**G**	**C**	**A**	C	G	G	T
		G	**T**	**C**	**G**	**T**				

Exception	**Ea**	A	T	T	G	**A**	**G**	**T**	**G**	**T**
		T	A	A	C	**T**				

*^a^ n, terminal base pair.*

*^b^ X is any nucleotide; A, C, G, T.*

*^c^ Chain terminator.*

*^d^ Letter code that corresponds to faint or missing Ts in the sequencing reactions shown in **Figure 3**.*

*Bold indicates highly conserved sequence.*

#### Increased incorporation of fluorophore-labeled nucleotides

The L412M-DNA polymerase has increased ability to incorporate modified nucleotides compared to the wild type T4 DNA polymerase, exonuclease-deficient T4 DNA polymerases and other mutant T4 DNA polymerases and DNA polymerases from other organisms (Goodman and Reha-Krantz, 1999). Reactions showing incorporation and extension of a variety of modified nucleotides with the L412M- and exonuclease-deficient L412M-DNA polymerase are shown in US patent 5945312. The modified nucleotides tested include, but are not limited to rhodamine-dUTP, fluorescein-dUTP, rhodamine-dCTP, biotin-dCTP and DIG-dCTP. For all modified nucleotides, the L412M-DNA polymerase performed significantly better than the wild type T4 DNA polymerase or DNA polymerases from other organisms. The T4 L412M-DNA polymerase was used to prepare fluorescently labeled DNA with one or two fluorophore-labeled bases for proof-in-principle testing of DNA sequencing by exonuclease digestion proposed by Keller and colleagues (Goodwin et al., 1995; Werner et al., 2003).

### THE PHAGE T4 EXONUCLEASE-DEFICIENT L412M-DNA POLYMERASE AS A DNA SEQUENCING ENZYME: FIDELITY^TM^

**FIGURE 5:**
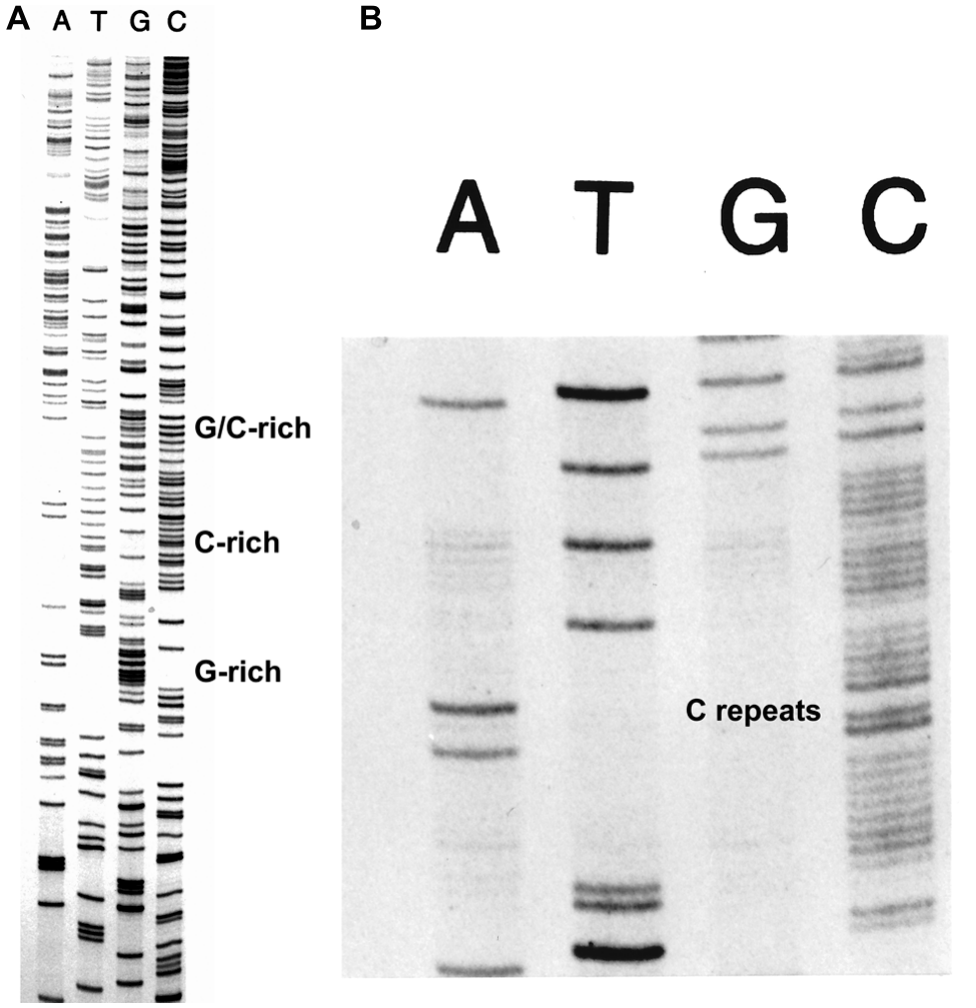
**Sequencing difficult DNAs with FIDELITY^TM^**. The combination of the exonuclease-deficient L412M-DNA polymerase, the T4 clamp and clamp loading proteins, and the T4 single-stranded DNA binding protein provides the means to sequence difficult DNAs with high G+C content **(A)** and tracks of mononucleotide repeats **(B)**.

The FIDELITY^TM^ DNA sequencing kit (ONCOR, Gaithersburg, MD, USA) was developed for routine DNA sequencing, but especially for sequencing difficult DNA sequences. The use of the exonuclease-deficient L412M-DNA polymerase with increased intrinsic processivity plus the addition of T4 processivity factors and ssDNA binding protein provided the means to sequence all types of difficult DNA sequences with greater success than achieved by other methods as judged by testimonials and applications, for example, see Devireddy and Jones (1998) and Tullius et al. (2001). An example of routine DNA sequencing is shown in **Figure 3** and examples of sequencing difficult DNAs are shown in **Figure 5**. Note the clean sequence for the G+C-rich template (**Figure 5A**) and for a template with several tracks of C repeats (**Figure 5B**).

### USING GENETIC APPROACHES TO IDENTIFY “BIOTECH” DNA POLYMERASES: *IN VIVO* AND *IN VITRO* SELECTION METHODS

Genetic selection methods are powerful tools to identify mutant DNA polymerases on the basis of phenotype without the need to have a complete understanding of structure or function. The advantage of genetic selection strategies is that if the stringency is sufficiently strong, then only mutants with the desired phenotype will survive. This expedites “finding a needle in a haystack.” The phage T4 L412M-DNA polymerase was the result of two consecutive genetic selection schemes.

First, a genetic selection was carried out for the identification of mutant T4 DNA polymerases with a strong mutator phenotype. Details for the selection of mutator T4 DNA polymerases (Reha-Krantz et al., 1986; Reha-Krantz, 1988) and a similar strategy for mutator yeast DNA polymerase δ mutants (Murphy et al., 2006) are described. Several mutator T4 DNA polymerases were identified, but not the L412M-DNA polymerase because this mutant replicates DNA with relatively high fidelity as discussed (**Table 2**). For many isolates there was more than one mutation in the DNA polymerase gene. Single mutant strains had to be constructed in order to determine if one or more mutations were required to confer the mutator phenotype and to rule out contributing mutations in other genes. For one isolate, called *mel5*, there were 11 mutations in the DNA polymerase gene, but only the mutation encoding the D131G amino acid substitution was required for mutator activity (Reha-Krantz, 1988). The D131G-DNA polymerase has strongly reduced ability to form exonuclease complexes and, as a consequence, has severely reduced 3′→5′ exonuclease activity (Baker and Reha-Krantz, 1998).

Why were there so many mutations in the *mel5* strain? One possibility is that error-prone replication by mutator DNA polymerases created many mutations within the DNA polymerase gene and in other genes during propagation, but a more likely explanation is that additional DNA polymerase mutations were *selected* to temper the mutational burden produced by the D131G amino acid substitution. This proposal is supported by the presence of a mutation in the *mel5* strain that encodes the I417V substitution in Motif A (**Table 1**). The I417V-DNA polymerase has an antimutator phenotype, increased proofreading, and low intrinsic processivity (Reha-Krantz and Nonay, 1994; **Figure 1** and **Table 2**), activities which would help to maximize residual exonuclease activity in the presence of the D131G substitution and increase replication fidelity. These activities, however, would also help to improve maturation of Okazaki fragments. Okazaki fragment maturation requires the coordinated action of DNA polymerase 3′→5′ exonuclease activity with a 5′→3′ exonuclease, ExoI/RNaseH in T4 and Rad27 in yeast, to create a nick that can be sealed by DNA ligase (Jin et al., 2004; reviewed by Reha-Krantz, 2010). In the absence of DNA polymerase 3′→5′ exonuclease activity, the DNA polymerase catalyzes strong displacement synthesis at junctions between Okazaki fragments that creates 5′→3′ flaps that prevent ligation unless the flap is removed. Persisting unjoined Okazaki fragments are dangerous because strand discontinuities result in double strand breaks in the next replication cycle. Thus, there are at least two strong selective pressures exerted to decrease the negative effects of reduced DNA polymerase proofreading.

The second selection to identify the L412M-DNA polymerase was to select for suppressors of the low processivity and high proofreading activity of the I417V-DNA polymerase (**Table 1** and **Figure 1**). One consequence of low processivity is the need for high dNTP pools to sustain replication. The I417V-DNA polymerase could not replicate DNA when the dGTP pool was reduced, which allowed for selection of second site mutations that allowed replication under low dGTP conditions (Reha-Krantz and Nonay, 1994; Stocki et al., 1995; Li et al., 2010). Mutations encoding the L412M substitution in Motif A and elsewhere in the DNA polymerase gene were identified (Stocki et al., 1995; Li et al., 2010). Thus, the mutation encoding the L412M substitution and functionally equivalent amino acid substitutions are expected to be identified as suppressors of mutant DNA polymerases with low processivity.

Selection schemes can also be done *in vitro* to identify mutant DNA polymerases with desired properties for biotech applications. Bourn et al. (2011) describe an *in vitro* strategy beginning with a chimeric DNA polymerase derived from *Pyrococcus kodakaraensis* and *Pyrococcus furiosus*, which was named Kofu DNA polymerase. Several mutant DNA polymerases with increased processivity and other desirable features were identified, but many had multiple mutations as we observed for *in vivo* selections with the T4 DNA polymerase. There are no reports to link amino acid substitutions in the Kofu DNA polymerase with specific polymerase properties and the amino acid substitutions in the DNA polymerase marketed as KAPA HiFi DNA polymerase by KAPA Biosystems Ltd., South Africa, are proprietary. Bourn et al. (2011) suggest, however, that advantageous properties may require the combined action of several amino acid substitutions. None of the amino acids disclosed, however, are in Motif A, but there could be amino acid substitutions in other DNA polymerase regions that affect processivity, for example see Stocki et al. (1995), Reha-Krantz and Wong (1996), and Li et al. (2010). While our genetic selections were done *in vivo*, *in vitro* strategies similar to Bourn et al. (2011) can be envisioned that exploit sensitivities to dNTP pool concentrations, PAA or PPi can be envisioned.

We have applied information learned about T4 DNA polymerase function to other DNA polymerases, namely yeast DNA polymerases alpha and delta. Amino acid changes in the yeast DNA polymerases that are analogous to the L412M substitution in the T4 DNA polymerase produce PAA-sensitivity (**Table 1**; Li et al., 2005). Because PAA-sensitivity is correlated with increased intrinsic processivity, the mutant yeast DNA polymerases are also predicted to have increased processivity; however, the mutant yeast DNA polymerases are more error prone than the L412M-DNA polymerase (**Table 2**; Niimi et al., 2004; Li et al., 2005). The differences in replication fidelity for the T4 and yeast DNA polymerases suggest differences in the equilibria between Pre- and Post-T complexes and in forming exonuclease complexes. However, fine tuning this balance can be achieved by testing other amino acid substitutions in Motif A and by combining amino acid substitutions. For example, the phage T4 L412M/I417V-DNA polymerase is PAA-sensitive, although less than the L412M-DNA polymerase, but the double mutant has an antimutator phenotype and slightly increased proofreading compared to the wild type T4 DNA polymerase (**Tables 1 and 2**). Thus, single or multiple amino acid changes within the Motif A sequence of DNA polymerases in general may produce mutant DNA polymerases with the optimal balance of intrinsic processivity and proofreading that will allow replication of difficult DNA sequences and increased utilization of modified nucleotides while maintaining replication fidelity.

## DISCUSSION

The phage T4 exonuclease-deficient L412M-DNA polymerase is proven to be an excellent DNA sequencing enzyme especially when combined with processivity factors (clamp and clamp loaders) and ssDNA binding protein; this is the basis of FIDELITY^TM^. All DNAs tested, including DNAs with long stretches of mono- and di-nucleotide repeats and high A+T- or G+C-content are sequenced cleanly (**Figure 5**; S. Woodgate personal observations). While the T4 L412M-DNA polymerase remains an attractive candidate as a DNA sequencing enzyme, other DNA polymerases may have additional desirable properties that would profit by modifications to the Motif A sequence. Indeed, in addition to our studies of Motif A in phage T4 and yeast DNA polymerases (Reha-Krantz and Nonay, 1994; Li et al., 2005; **Table 1**), Motif A in family B and A DNA polymerases is surprisingly amenable to engineering despite being a conserved sequence (**Table 1**; Patel and Loeb, 2000; Niimi et al., 2004; Venkatesan et al., 2006, 2007). Our studies demonstrate that Motif A functions in determining intrinsic processivity for the phage T4 DNA polymerase (**Figure 1**) and, by extrapolation, to other DNA polymerases. The level of intrinsic processivity determines the ability of DNA polymerases to replicate difficult DNA sequences and to incorporate modified nucleotides; these properties are maintained even when dNTP pools are reduced for the L412M-DNA polymerase (**Figure 1**). Different amino acid substitutions and combinations of substitutions in Motif A allow fine-tuning to achieve the optimal balance of intrinsic processivity for the replication of difficult DNA templates while maintaining high replication fidelity; these are highly desirable properties for sequencing, amplification and labeling technologies. We focus here on Motif A because this motif is conserved in all DNA and RNA polymerases, which means that site-directed mutagenesis of Motif A can be used to identify mutant DNA polymerases with increased intrinsic processivity that still retain adequate proofreading activity. However, other DNA polymerase regions also affect intrinsic processivity (Reha-Krantz et al., 1993; Stocki et al., 1995; Li et al., 2010) and, thus, are attractive secondary mutational targets.

Increased intrinsic processivity increases pyrophosphorolysis, but methods were shown to curb pyrophosphorolysis activity of the exonuclease-deficient L412M-DNA polymerase by the addition of iPPase or divalent metal ions, Mn^2+^ or Ca^2+^ (**Figure 3**). In the absence of these agents, pyrophosphorolysis under DNA sequencing conditions displayed sequence specificity. Pyrophosphorolysis was detected only for a subset of sequencing products terminated with T (**Figure 3**). Although pyrophosphorolysis by the T4 wild type and L412M-DNA polymerases was higher for DNA substrates with A+T- compared to G+C-rich primer terminal regions, the sequence specificity observed under DNA sequencing conditions was specific for primer-ends with the T-terminating nucleotide plus either a C_template_G_primer_ base pair in the -1 position or a G_template_C_primer_ base pair in the -2 position or a C_template_G_primer_ in the -1 and a G_template_C_primer_ in the -2 position (**Table 3**). Dinucleotide repeats may also sensitize terminal Ts, see the pyrophosphorolysis sensitive site Ea in **Figure 3** and **Table 3**. The preference of pyrophosphorolysis for A+T-rich primer-terminal regions is expected for the T4 DNA polymerase, because A+T-rich primer-termini favor formation of Pre-T complexes (Hariharan and Reha-Krantz, 2005), which can bind PPi and PPi-like antiviral drugs (**Figure 2**). The sequence-dependence for pyrophosphorolysis under DNA sequencing conditions must also indicate sequences that favor Pre-T complexes, but the mechanism is unclear. Unfortunately, while there are numerous structural studies of the RB69 DNA polymerase (for example Franklin et al., 2001; Hogg et al., 2004; Wang et al., 2011), which is a close relative of the phage T4 DNA polymerase (Hogg et al., 2006), the primer-template DNAs used in crystallography did not have the pyrophosphorolysis-sensitive sequences indicated in **Table 3**; thus, a direct test of DNA polymerase interactions with the pyrophosphorolysis sequences has not been done. However, the DNA substrate used to capture a mutant RB69 DNA polymerase with the PPi analog, PFA (foscarnet), was A+T-rich except for the terminal acyclo guanine (Zahn et al., 2011), which is consistent with our data that demonstrate increased pyrophosphorolysis with A+T-rich DNAs.

We propose that the sequence specificity for the pyrophosphorolysis reaction under DNA sequencing conditions is caused by subtle interactions of the DNA polymerase with a dynamic primer-terminal region that breathes more or less depending on the DNA sequence, but we do not rule out the possibility of base recognition, especially for bases in the +1 and +4 positions in the template strand (**Table 3**). Breathing in the terminal region is proposed to explain why A+T-richness favors proofreading by increasing the strand separation needed to form exonuclease complexes (for example, see Bloom et al., 1994 and Bessman and Reha-Krantz, 1977); we suggest here that breathing also affects the rapid equilibrium between Pre- and Post-T complexes and increased breathing favors Pre-T complexes. Breathing has been detected at the primer-terminal junction by fluorescence studies that demonstrate measurable unwinding ∼2 base pairs into the duplex region (Jose et al., 2009), which may be exacerbated by DNA polymerase interactions. But how does the DNA polymerase distinguish between opting for pyrophosphorolysis compared to proofreading? Proofreading a chain-terminated primer-end may always be preferred, but the DNA polymerase in sequencing reactions is exonuclease-deficient which allows detection of pyrophosphorolysis, but pyrophosphorolysis is not simply a default pathway because sequence specificity is observed. The pyrophosphorolysis-sensitive DNA sequences (**Table 3**) must stabilize formation of stable Pre-T complexes (reduce dissociation) at the expense of Post-T complexes. In other words, sequences that promote pyrophosphorolysis must hinder translocation to form Post-T complexes.

A critical step in translocation may involve interactions of the DNA polymerase at the -1 position in the primer-terminal region, using the naming system indicated in **Table 1** with “n” designating the terminal base pair. Structural studies show that a conserved lysine residue intercalates into the primer-terminal region at the -1 position of ternary complexes formed with the RB69 DNA polymerase (Franklin et al., 2001), but in complexes trapped with the PPi mimic PFA (Zahn et al., 2011), translocation was blocked after nucleotide incorporation and lysine intercalation did not change, which meant intercalation was observed at the -2 position. Thus, lysine intercalation advances step wise after each nucleotide is incorporated with intercalation at the -2 base pair position in Pre-T complexes before translocation and advancing to the -1 base pair position to form Post-T complexes. The -1 and -2 positions are also critical for promoting pyrophosphorolysis; a G_template_C_primer_ base pair is observed in the -2 position of sequences that promote pyrophosphorolysis (sequence 1) unless there is a C_template_G_primer_ base pair in the -1 position (sequence 2) or two GC and CG base pairs are observed in the -2 and -1 positions, respectively (**Table 3**). If localized breathing in the primer-terminal region is the critical factor for forming stable Pre-T complexes, disfavoring exonuclease complexes, and hindering translocation to form Post-T complexes, then the requirement of GC and CG base pairs at the -2 and -1 positions suggests that GC base pairs impart stability that reduces breathing and strand separation needed to form exonuclease complexes. But what hinders formation of Post-T complexes? One possibility may reflect the effect of duplex stability on the ability of the conserved lysine residue to intercalate into duplex DNA if the ease of intercalation is affected by A+T/G+C-richness. Another possibility may be that sequences which promote pyrophosphorolysis cause a slight distortion of the DNA helix. This possibility is suggested because not any GC base pair is sufficient, but only a G_template_C_primer_ base pair in the -2 position or a C_template_G_primer_ base pair in the -1 position, not the reverse Watson-Crick base pairs. There is also specificity for A_template_T_primer_ or C _template_G_primer_ base pairs in the -3 position. While base recognition has not been ruled out, subtle physical changes in the primer-terminal region appear to affect the equilibria between Pre- and Post-T complexes and exonuclease complexes. This suggestion is in keeping with trying to understand the underlying mechanism to explain how the conservative L412M substitution in the T4 DNA polymerase has such profound effects on processivity and sensitivity to the PPi-mimic PAA (**Figure 1**; Reha-Krantz and Nonay, 1994) while the L412I substitution also has profound effects, but in the opposite direction. Structural studies of the analogous RB69 L415M-DNA polymerase of Post-T ternary complexes with dNTP (Xie et al., 2013) or Pre-T complexes trapped with PFA (Zahn et al., 2011) do not shed light on mechanism, which then leaves open the possibility that rapidly changing conformations not yet captured in structural studies are behind the observed characteristics of the T4 L412M- and L412I-DNA polymerases.

Future studies are needed to determine if the pyrophosphorolysis-sensitive sequences described in **Table 3** promote pyrophosphorolysis in the absence of a chain-terminating T or if the chain-terminator contributes to the observed sequence specificity. DNA binding studies with the primer-templates described in **Table 3** are also needed. Studies are also needed to explore the possibility that bases in the +1 and +4 positions in the template are recognized by the T4 DNA polymerase. The ability of “A” in the +4 position in the template strand to prevent pyrophosphorolysis suggests that the DNA polymerase can detect sequence. Interestingly, the Pfu DNA polymerase detects deaminated bases in the +4 position as part of an apparent error avoidance mechanism (Connolly, 2009). The sequence specificity of the pyrophosphorolysis reaction suggests possible implications for antiviral drug therapy. For example, is the sensitivity of herpes viral DNA polymerases to PPi analogs, PAA/PFA/foscarnet, dependent on DNA sequence? See additional discussion by Li et al. (2010).

In conclusion, amino acid substitutions in the phage T4 DNA polymerase Motif A increase or decrease intrinsic processivity by altering the equilibria between Pre- and Post-T complexes and formation of exonuclease complexes. Amino acid changes in Motif A affect intrinsic processivity by increasing the stability of Pre-T complexes. Increased stability of Pre-T complexes means increased ability to replicate difficult DNA sequences and to incorporate modified nucleotides. Motif A is an attractive target for engineering “biotech” DNA polymerases because intrinsic processivity can be fine-tuned by using different amino acid substitutions and combinations of amino acid substitutions to optimize replication of difficult DNA sequences and to enhance the ability to use modified nucleotides while maintaining replication fidelity. Another important take-home message is to acknowledge that DNA and DNA polymerase-DNA interactions are dynamic, especially in the primer-terminal region (Hariharan and Reha-Krantz, 2005; Hariharan et al., 2006; Jose et al., 2009). Lastly, DNA polymerases may have more ability to detect specific sequences than previously recognized.

## AUTHOR CONRIBUTIONS

Linda J. Reha-Krantz was responsible for organizing and writing the initial manuscript and for providing new data. Sandra Woodgate and Myron F. Goodman added intellectual content and critically evaluated the manuscript. Sandra Woodgate developed the FIDELITY^TM^ DNA sequencing kit in collaboration with Myron F. Goodman and Linda J. Reha-Krantz and she provided data on the pyrophosphorolysis reaction under DNA sequencing conditions. All authors agree to be accountable for all aspects of the work presented.

## Conflict of Interest Statement

The authors declare that the research was conducted in the absence of any commercial or financial relationships that could be construed as a potential conflict of interest.
